# A Targeted Genetic Modifier Screen Links the SWI2/SNF2 Protein Domino to Growth and Autophagy Genes in *Drosophila melanogaster*

**DOI:** 10.1534/g3.112.005496

**Published:** 2013-05-01

**Authors:** Matt Hyoung Kwon, Heather Callaway, Jim Zhong, Barry Yedvobnick

**Affiliations:** Biology Department, Emory University, Atlanta, Georgia 30322

**Keywords:** chromatin, gene regulation, cell proliferation, autophagy, Notch signaling

## Abstract

Targeted genetic studies can facilitate phenotypic analyses and provide important insights into development and other complex processes. The SWI2/SNF2 DNA-dependent ATPase Domino (Dom) of *Drosophila melanogaster*, a component of the Tip60 acetyltransferase complex, has been associated with a wide spectrum of cellular processes at multiple developmental stages. These include hematopoiesis, cell proliferation, homeotic gene regulation, histone exchange during DNA repair, and Notch signaling. To explore the wider gene network associated with Dom action, we used RNAi directed against *domino* (*dom*) to mediate loss-of-function at the wing margin, a tissue that is readily scored for phenotypic changes. *Dom* RNAi driven through *GAL4-UAS* elicited dominant wing nicking that responded phenotypically to the dose of *dom* and other loci known to function with *dom*. We screened for phenotypic modifiers of this wing phenotype among 2500 transpositions of the *EP P* element and found both enhancers and suppressors. Several classes of modifier were obtained, including those encoding transcription factors, RNA regulatory proteins, and factors that regulate cell growth, proliferation and autophagy, a lysosomal degradation pathway that affects cell growth under conditions of starvation and stress. Our analysis is consistent with prior studies, suggesting that Dom acts pleiotropically as a positive effector of Notch signaling and a repressor of proliferation. This genetic system should facilitate screens for additional loci associated with Dom function, and complement biochemical approaches to their regulatory activity.

Genetic interaction screening is a powerful tool for the analysis of complex processes. In cases where a gene acts throughout development, targeting loss of function to a later stage can abrogate early lethality and allow application of effective genetic strategies. In *Drosophila*, targeted genetic screening has been used to study the genetics of key developmental pathways operating in specific tissues at particular times (Zhong and Yedvobnick 2009). Such screening often uses the yeast *GAL4-UAS* system to express high levels of normal or mutated versions of genes in a defined fashion. Phenotypes derived from such constructs allow screens for modifiers, thereby expanding the known set of genes that contribute to a pathway (Brand and Perrimon 1993; Rorth 1996). For example, by targeting the wing margin and eye, these methods were used to identify novel genes that contribute to the Notch pathway, a major signaling system of metazoans (Hall *et al.* 2004; Alexander *et al.* 2006; Kankel *et al.* 2007; Shalaby *et al.* 2009). These screens, among others (Mummery-Widmer *et al.* 2009, Yatim *et al.* 2012), have revealed a wide network of loci that impinge on Notch signaling at numerous levels. One identified locus named *domino* (*dom*), originally linked to hematopoiesis and homeotic gene repression (Braun *et al.* 1997, Ruhf *et al.* 2001), also interacts genetically with Notch during wing margin formation (Hall *et al.* 2004). Subsequent studies further elaborated *dom*’s role in Notch pathway regulation (Eissenberg *et al.* 2005; Gause *et al.* 2006) and other processes including exchange of phosphorylated histone H2Av as part of the Tip60 acetyltransferase complex (Kusch *et al.* 2004), germline and somatic stem cell self-renewal (Xi and Xie 2005), and repression of E2F responsive loci (Lu *et al.* 2007). The *dom* gene sequence predicts two major proteins of the SWI2/SNF2 class of DNA-dependent ATPases, implicating *dom* in gene regulation at the level of chromatin modification/nucleosome remodeling (Ruhf *et al.* 2001). Dom proteins are widely expressed in embryos and imaginal discs, and the sequence is highly conserved (Ruhf *et al.* 2001; Kusch *et al.* 2004; Eissenberg *et al.* 2005). Moreover, alleles of *dom* result in larval or pupal lethality (Ruhf *et al.* 2001). We reasoned that a multifunctional chromatin remodeling protein that acts at the wing margin would be a practical choice for a targeted genetic modifier screen. Genetic changes that interact with a *dom*-associated wing phenotype should allow a sensitive screen for genes functioning with, or regulated by Dom. Therefore, we constructed a strain that expresses RNAi directed against *dom* transcripts at the wing margin. This strain shows a dominant wing phenotype that can be modified through changes in expression of loci known to interact with *dom*. Using this strain in a transposon-based genetic screen we obtained several classes of modifier, including those encoding transcription factors, RNA-binding proteins, and several proteins associated with growth regulation and autophagy. These modifiers link Dom function to cell proliferation, as suggested by others (Braun *et al.* 1997; Ruhf *et al.* 2001; Lu *et al.* 2007). Thus, this preliminary screen for modifiers of a *dom* wing phenotype indicates that it is a reliable method to further dissect *dom* function.

## Materials and Methods

### *Drosophila* strains

Strains were obtained from the following laboratories: *C96-GAL4* (G. Boulianne, University of Toronto, Toronto, ON, Canada), *blk-GAL4* l (M. Hoffman, University of Wisconsin, Madison, WI), *vg-GAL4*, *pnr-GAL4*, *nd^1^*, *UAS-N* (activated Notch, weakly expressing strain, S. Artavanis-Tsakonas, Harvard Medical School, Boston, MA), *UAS-Wdb* (J. Jia, University of Kentucky, Lexington, Kentucky), *pumilio^01688^* (H. Lin, Yale University, New Haven, CT), *atg1 = Unc51^3^* and *UAS-Unc51*(8) (T. Tomoda, Beckman Research Institute, Duarte, CA), *ptc-GAL4*, *Delta^BX9^* (M. Muskavitch, Boston College, Chestnut Hill, MA), *mam^N2G^* (J. Campos-Ortega, deceased), *dom RNAi* (*3*) and *UAS-DomB* (M. Ruhf, University of Cincinnati, Cincinnati, OH), *dom^EP2371^* (Exelixis, San Francisco, CA), *cyclin E^AR95^/Cy* and *UAS-Rbf-280* (K. Moberg, Emory University, Atlanta, GA). *C96-MamH* was described previously (Helms *et al.* 1999).

The following strains were obtained from the Bloomington (BL) Stock Center: *P{EP}peb^EP55^* (5358), *y^1^ w^1^*; *Ki^1^ P{Δ2-3}99B* (4368), *y^1^ w^67c23^*; *P{lacW}dom^1^/CyO* (10767), y^1^ w*; *dom^3^/SM6a* (9260), y^1^ w*; *dom^9^/CyO*, *y^+^* (9261), *sno^e1^/FM7a* (8745), *y^1^ v^1^*; *P{TRiP.JF01502}attP2 dom* (31054), *y^1^ w^67c23^*; *P{EPgy2}lilli^11976^* (20719), *EcR^M554fs^/SM6b* (4894), *lilli^[A17-2]^ cn^1^ bw^1^/CyO* (5726), *y^1^ w^67c23^*; *P{EPgy2}emc^EY01657^/TM3*, *Sb^1^ Ser^1^* (20124); *emc[D] rho[ve-1] rs[2] st[1] bul[D]/TM1*(1032), w[*]; *lola[ORE119]/CyO* (28284), y^1^ w*; *P{UAST-YFP.RabX1.T19N}Pabp2^01^/SM5* (9838), *y^1^ w^67c23^*; *P{EPgy2}Tudor-SN^EY07875^* (17412), *w^1118^*; *Lk6^2^*/*TM6B*, *Tb^1^* (8707), *y^1^* w*; *P{UASp-YFP.Rab30.T21N}PP2A wdb^07^* (9813), *y^1^* w*; *P{UASp-YFP.Rab9Fb.D66L}PP2A wdb^19^*/*TM3*, *Sb^1^* (9844), *y^1^ v^1^*; *P{TRiP.JF02897}attP2 atg6* (28060), *y^1^ v^1^*; *P{TRiP.JF02787}attP2 atg7* (27707), *y^1^ v^1^*; P{TRiP.JF02895}attP2 e* atg8A /TM3, *Sb^1^* (28989), *y^1^ v^1^*; *P{TRiP.JF02706}attP2 atg8B* (27554), *y^1^ v^1^*; *P{TRiP.JF02704}attP2 atg12* (27552), *y^1^ v^1^*; *P{TRiP.JF02891}attP2 atg9* (28055), *y^1^ v^1^*; *P{TRiP.JF02703}attP2 atg5* (27551), *y^1^ v^1^*; *P{TRiP.JF03003}attP2 atg4* (28367), *y^1^ v^1^*; *P{TRiP.JF02786}attP2 atg2* (27706), *y^1^ v^1^*; *P{TRiP.JF02898}attP2 atg18* (28061), *y^1^ v^1^*; *P{TRiP.HM05150}attP2* PP2A *wdb* (28939), *tara^1^/TM3 Sb* (6403), *y^1^ v^1^*; *P{TRiP.JF03141}attP2/TM3*, *Sb^1^* PP2A *tws* (28714), *y^1^* sc* *v^1^*; *P{TRiP.HM05256}attP2* PP2A *wrd* (30512), *y^1^ v^1^*; *P{TRiP.JF02805}attP2* PP2A *mts* (27723), *y^1^ v^1^*; *P{TRiP.JF03316}attP2* PP2A 29B (29384), *y^1^ v^1^*; *P{TRiP.HM04075}attP2 CK1* (31763), and w*; *P{UAS-dco.K}4* (26274)*. cn^1^ E*(*Pc*)*^1^ bw^1^/SM5* (3056), *y^1^ w^1118^*; *P{lacW}MRG15^j6A3^/TM3*, *Sb^1^* (10290), *y^1^*; *P{SUPor-P}Nipped-A^KG10162^/CyO* (16514), *y^1^ w^67c23^*; *P{EPgy2}rept^EY12756^/TM3*, *Sb^1^ Ser^1^* (21384), *y^1^ v^1^*; *P{TRiP.HM05049}attP2 Tip60* (28563).

### Construction of *UAS-domR* plasmid

The forward primer GGGAGTCCGATGGTGAGTTA and reverse primer ACTTGCGCTCATTCATTGTG were used to amplify a 1057-bp segment of genomic DNA from the *dom* locus (genomic position 17215713–17216769). This segment spanned the 3′ end of exon 5, intron 5, and most of exon 6 (Ruhf *et al.* 2001) and was chosen to minimize nucleotide similarities to other genomic regions. The PCR product was cloned into *pCR2.1* (Invitrogen) and then subcloned into *SympUAST-w* (Giordano *et al.* 2002). The resulting plasmid *UAS-domR* was transformed into the germline by Genetic Services, Inc. Chromosome 3 inserts of *UAS-domR* and *C96-GAL4* were recombined to create the *C96-domR/TM3 Sb* strain. The *C96-GAL4* driver expresses across the dorsal-ventral wing margin of third-instar larvae (Gustafson and Boulianne 1996; Helms *et al.* 1999). The *C96-domR* chromosome is associated with a dominant but partially penetrant wing nicking phenotype described in *Results*.

### Generation of *EP* transpositions and scoring for modifiers

Mobilization of the X chromosome-linked *w****^+^****EP55* element via the transposase *P[ry+ Δ2-3] Ki^1^* (*99B*) was described in detail previously (Kazemi-Esfarjani and Benzer 2000; Alexander *et al.* 2006). After introduction of the transposase into *w^+^ EP* males, 3 males were mated in a vial with 5 white-eyed (*w^1118^*) females, which are homozygous for *w^−^* on the X chromosomes. Any male offspring from this cross exhibiting red eyes must have a transposition of the element from the X chromosome to an autosome or rarely to the Y chromosome. Non *Ki* flies with nonmottled red eyes were selected to eliminate the transposase and stabilize the insertion. Only a single transposition male from each vial was analyzed. Each male carrying a transposition was mated to *C96-domR/TM3 Sb* females, and progeny were scored for enhancement or suppression of the wing nicking phenotype. Modifiers were retested by crossing once again to *C96-domR*, then outcrossed to a balancer strain, and made homozygous or balanced in the case of recessive lethals. A subset of modifiers was mated to candidate mutations, including the Notch pathway loci *mastermind* and *Delta*. In some cases, modifiers were also mated with deficiencies covering *P* element hotspots to eliminate multiple alleles. Modifiers were also crossed with the *C96-GAL4* strain to rule out wing effects that were independent of *dom* RNAi. All modifiers described here exhibited no phenotypes with this control cross. Likewise, we did not observe significant interaction of the modifiers with a strain exhibiting a *GAL4*-driven eye phenotype, suggesting that the modifiers were not selected based on a strictly *GAL4*-dependent effect.

*EP* modifiers were scored as enhancers or suppressors of *C96-domR* based on the penetrance of wing nicking relative to that of the *w^1118^* control crosses, rather than the severity of wing blade loss or nicking. Crosses of *C96-domR* to *w^1118^* produce a 57% penetrant phenotype (57% of wings exhibit at least one anterior margin nick). Each *EP* test against *C96-domR* included *w^1118^* controls, and wing nicking percentages were normalized to the 57% control value between experiments. For the twelve *EP* lines described here, the differences in penetrance between *C96-domR* and that of *w^1118^* were highly significant (*P* < 0.001, chi-square test) (Table 2).

### Generation of *dom^EP2371^* revertant

*w^+^dom^EP2371^* is a recessive lethal, *P element*-induced allele of *dom* that does not complement the *dom^1^* or *dom^3^* allele. *w^+^dom^EP2371^* was mobilized with transposase and *w*^−^ revertants isolated. Each revertant was assayed for restoration of *dom* function through crosses to *dom^1^* and *dom^3^*. *w^−^dom^EP2371Rev^* complemented both alleles.

### Identification of genomic sequence flanking *EP*s through inverse PCR

Procedures for the isolation of genomic DNA, restriction digestion, ligation, and inverse PCR were derived from the BDGP Website (http://www.fruitfly.org/about/methods/inverse.pcr.html). PCR products were purified with a QIAquick column (Qiagen), quantified on gels, and sequenced commercially by Macrogen. Sequence data were analyzed via FlyBlast (http://flybase.net/blast), and the *EP UAS*-driver oriented to genomic sequences by using the FlyBase Genome Browser.

### Antibody staining of third instar larval wing discs

The following protocol (Brennan *et al.* 1998) was followed for the fixation, staining, and washing of imaginal discs. Discs were dissected in 0.1 M sodium phosphate buffer (pH 7.4), fixed (2% paraformaldehyde, 75 mM lysine, 0.25% sodium periodate, 50 mM sodium phosphate pH 7.4) for 45 min, and washed (0.1 M sodium phosphate, 0.1% Triton X-100, pH 7.4). They were blocked in wash buffer containing 10% normal goat serum (NGS) and incubated with primary antiserum for 60 min at 37°. The discs were then washed twice, blocked, and incubated with secondary antibodies overnight at 4°. After three final washes, the discs were mounted in Slowfade (Molecular Probes). Images were obtained using an MRC 1024 model confocal microscope (Bio-Rad) and assembled with Photoshop software (Adobe). The following antibodies and dilutions were used: Wg (1:20 dilution, mouse monoclonal; Developmental Studies Hybridoma Bank), Cut (1:20 dilution, mouse monoclonal; Developmental Studies Hybridoma Bank). Alexa 488 secondary antibodies were obtained from Invitrogen and used at a dilution of 1:200). Discs derived from *dom^9^* and *dom^3^* larvae were selected opposite a green fluorescent protein (GFP)-marked balancer.

### Mounting of wings

Wings were dehydrated in isopropanol, mounted in Euparol, and photographed under a dissecting microscope (Hall *et al.* 2004).

## Results

### Generation of a *domino* RNAi-based dominant wing phenotype

**Figure 1:**
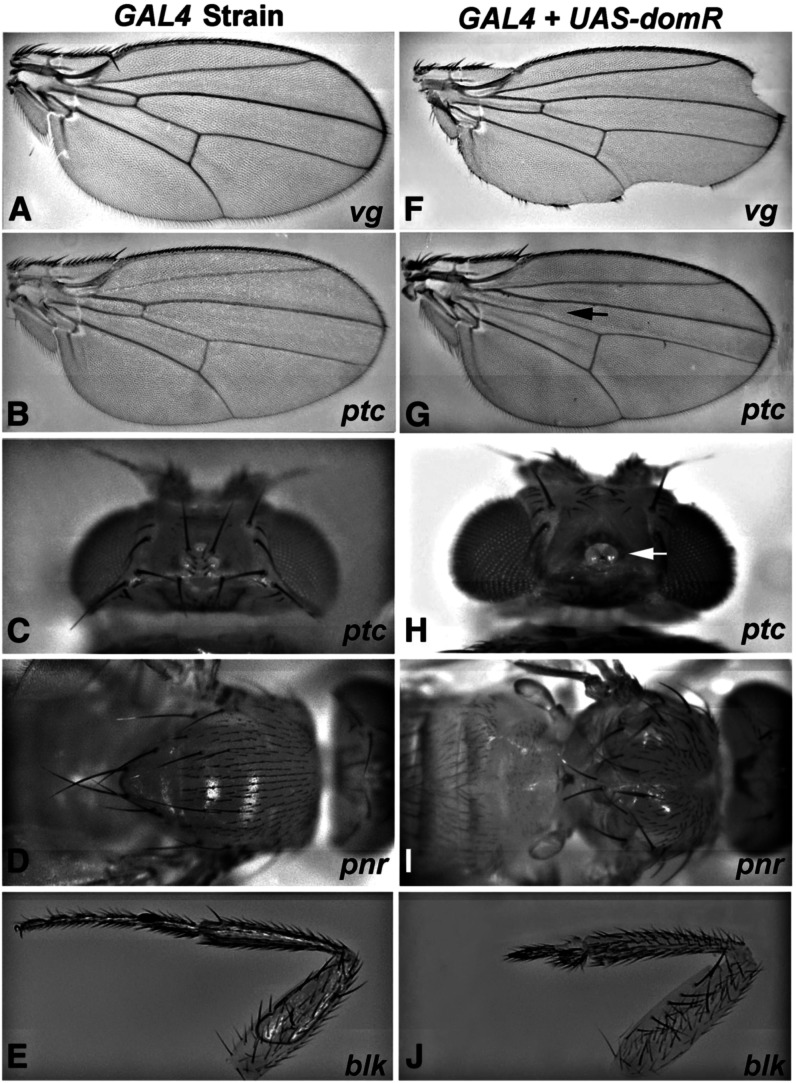
*GAL4*-driven *dom-RNAi* construct produces adult phenotypes. (A–E) Control tissues derived from crosses of *GAL4* lines to *w^1118^*; (F–J) results when the same *GAL4* lines were crossed with the *UAS-domR* RNAi construct. (A and F) *vg-GAL4* wing nicks; (B and G) *ptc-GAL4* loss of anterior crossvein (arrow); (C and H) *ptc-GAL4* loss of head bristles and fusion of ocelli (arrow); (D and I) *pnr-GAL4* notum fusion incomplete; (E and J) *blk-GAL4* loss of distal leg segments.

We cloned a segment of *dom* (Ruhf *et al.* 2001) into a modified *pUAST* vector (*sympUAST*) to allow symmetrical transcription of sequences cloned in between two sets of *UAS* (Giordano *et al.* 2002). The targeted *dom* sequence (see *Materials and Methods* ) is common to both major transcripts (Ruhf *et al.* 2001) and was selected to minimize matches to other genomic sites, as well as CAN repeats (Ma *et al.* 2006). We crossed transformants of the *sympUAST* construct (*domR*) with a panel of *GAL4* lines that drive expression in various imaginal tissues. As shown in Figure 1, the *domR* construct elicits prominent phenotypic effects when driven in the wing, head, notum, and legs (Figure 1, F–J) compared to the controls (Figure 1, A–E) expressing *GAL4* alone. Wing nicking via *vg-GAL4* is consistent with our identification of *dom* as a Notch modifier (Hall *et al.* 2004). These data are also consistent with the reported expression of *dom* in imaginal discs (Ruhf *et al.* 2001).

**Figure 2:**
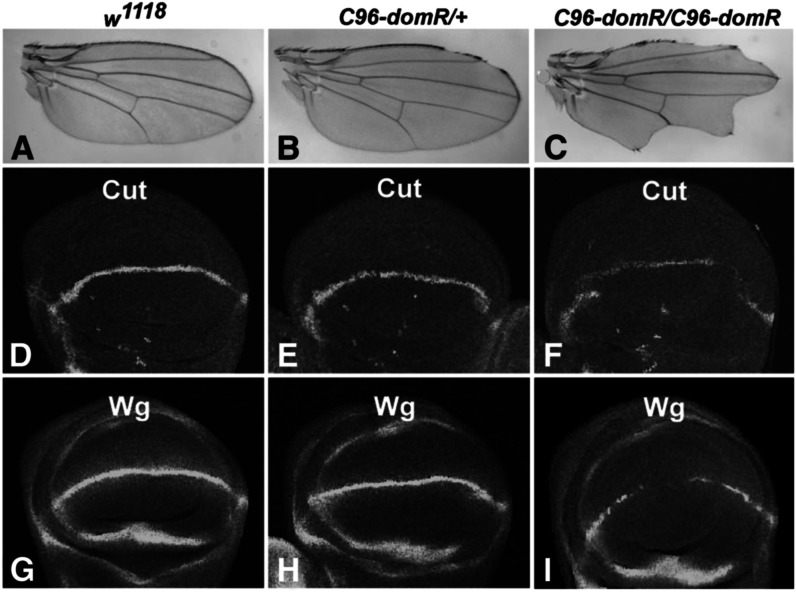
*dom* RNAi expression affects wing margin formation. Panels show wings or wing imaginal discs stained for Cut or Wg from *w^1118^* (A, D, and G); *C96-domR* heterozygotes (B, E, and H); and *C96-domR* homozygotes (C, F, and I). *C96-domR* is a recombinant chromosome containing both *C96-GAL4* and *UAS-domR* RNAi transgenes.

We also observed *domR* wing effects when driven with *C96-GAL4* (Helms *et al.* 1999), though to a lesser extent than with *vg-GAL4*. *C96-GAL4* and *domR* transgenes were recombined on chromosome 3 (*C96-domR*) to perform genetic tests (Figure 2). The predominant effects in *C96-domR* heterozygotes occur along the anterior margin, whereas effects are widespread in homozygotes (Figure 2, B and C). We assayed the wing margin protein markers Cut and Wg (Helms *et al.* 1999) in wing discs and observed minor stain depressions in heterozygotes (Figure 2, E and H) and substantial loss of staining in homozygotes (Figure 2, F and I).

**Figure 3:**
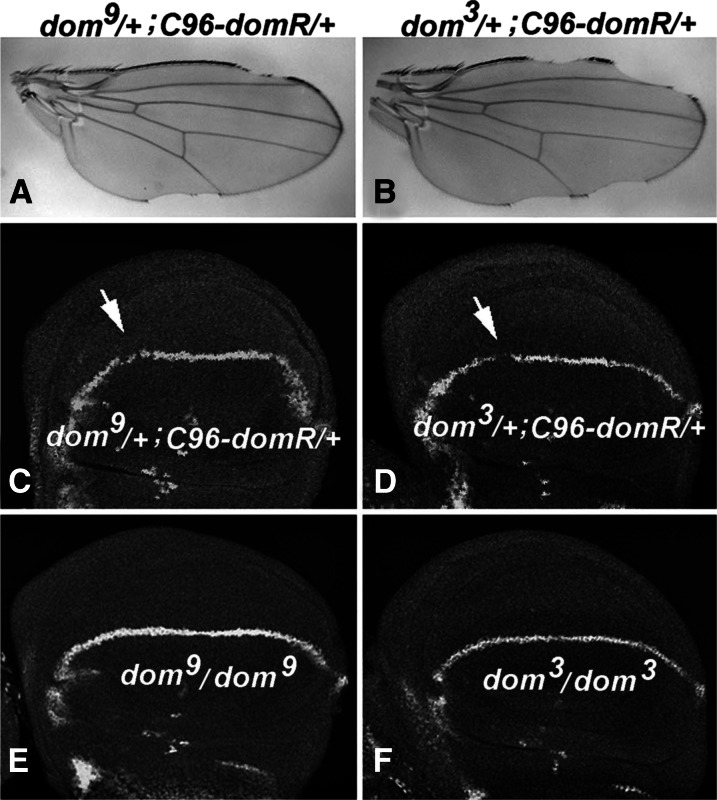
Effects of *domR* relative to canonical *dom* alleles. (A and C) Adult wing and wing disc derived from *dom^9^*/+; *C96-domR/+* heterozygotes. (B and D) Same, derived from *dom^3^*/+; *C96-domR/+* heterozygotes. Arrows show future anterior wing margin with gaps in staining. (E and F) Wing disc tissues from *dom^9^/dom^9^* homozygotes and *dom^3^/dom^3^* homozygotes, respectively. Wing discs were stained for Cut expression.

We also measured the effectiveness of *domR* relative to that of canonical loss-of-function (LOF) *dom* alleles in down-regulating the Cut protein. The weak *dom^9^* allele is less severe than the moderate allele *dom* (Ruhf *et al.* 2001; and see Table 1), and neither allele produces a wing phenotype as a heterozygote after outcross to *w^1118^* (data not shown). As expected, transheterozygotes of *C96-domR* with *dom^9^* show a weaker phenotype than transheterozygotes of *C96-domR* with *dom^3^* (Figure 3, A and B). Staining of wing discs for these transheterozygotes reveals depressions in Cut expression, especially in sections of the future anterior margin (Figure 3, C and D). Homozygotes for *dom^9^* and *dom^3^* survive as larvae (Figure 3, E and F) and show wing margin levels of Cut that are higher than the transheterozygotes of *C96-domR*. Thus, one copy of the *C96-domR* construct appears to diminish *dom* function to a greater extent than these canonical alleles of *dom*, using Cut expression as the measure.

**Table 1:** Validation *C96-domR* RNAi phenotype

Tester Genotype	% of Nicked Wings	*N* Wings Scored
*w^1118^*	57%	1756
*dom^1^*	84%	492
*dom^3^*	98%	140
*dom^9^*	72%	116
*dom^2371^*	81%	296
*dom^2371rev^*	59%	318
*UAS-DomB*	20%	644
*dom RNAi 3*	79%	1139
*dom RNAi TRiP*	76%	939

Tester genotypes are transheterozygous for *C96-domR* chromosome. The *dom^2371rev^* revertant combined with *C96-domR* does not show wing nicking at a significantly higher frequency (59%) than *w^1118^* control combined with *C96-domR* (57%; *P* = 0.63, chi-square test). All remaining genotypes were highly significant (*P* < 0.001).

### Validation of *dom* RNAi Effects

To validate *domR* RNAi effects (Ma *et al.* 2006; Perrimon and Mathey-Prevot 2007) we performed a series of genetic tests (Table 1). The table shows the percent nicked wings in genotypes heterozygous for the *C96-domR* construct and various test chromosomes, including a *w^1118^* control. *C96-domR/w^1118^* heterozygotes exhibit a 57% penetrant wing nicking effect (57% of wings show at least one anterior nick). When transheterozygous with strong or moderate LOF alleles for *dom* the penetrance rises, for example, *dom^1^* (84%) and *dom^3^* (98%), whereas less enhancement is evident with a weak allele, for example, *dom^9^* (72%), that derives from the identical genetic background as *dom^1^* and *dom^3^*. The *P element* insertion allele *dom^2371^* also exhibits strong enhancement (81%), whereas a *dom^+^* revertant chromosome (*dom^2371Rev^*) eliminates the enhancement (59%). Moreover, when the normal Dom B (*UAS-DomB*) protein, which is highly expressed in imaginal discs (Ruhf *et al.* 2001), is driven along with *C96-domR*, significant rescue is observed, as the penetrance of wing nicking drops to 20%. We also examined two independent *dom* RNAi constructs for enhancement of the *C96-domR* phenotype (*dom RNAi 3* and *dom RNAi TRiP*), and both constructs produced significant enhancement. These data demonstrate that the *C96-domR* RNAi wing phenotype responds as expected to loss- or gain-of-function (GOF) for *dom*, verifying its effect on *dom* function.

**Figure 4:**
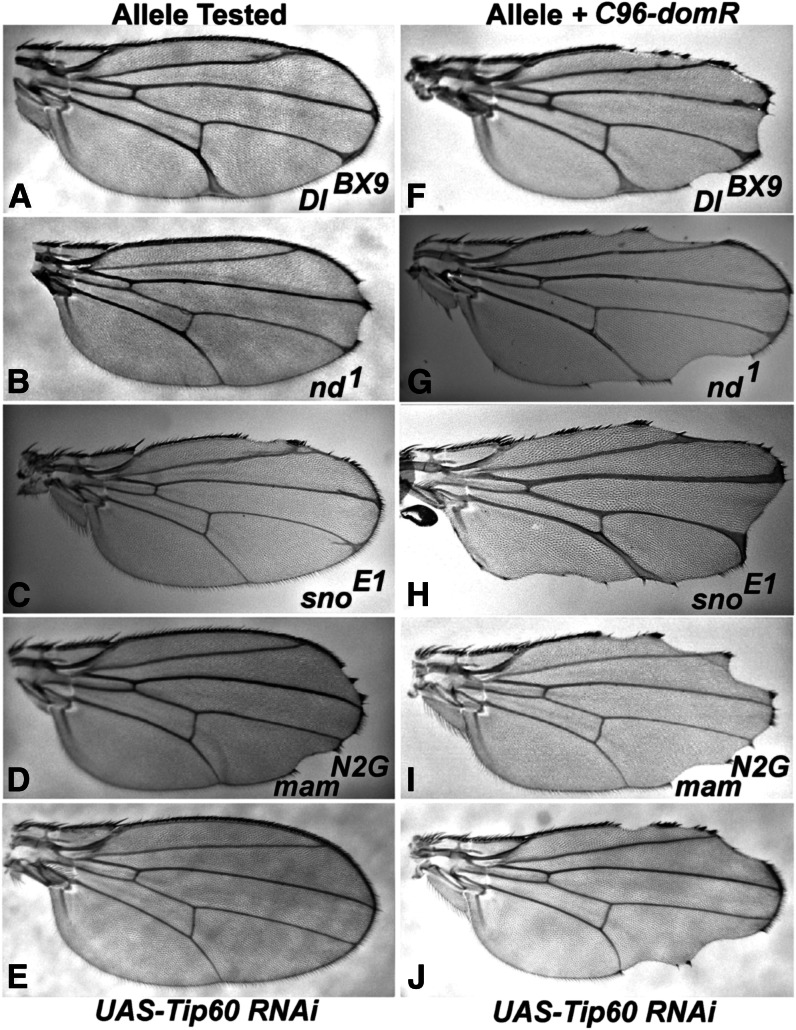
*dom* RNAi expression modifies *Notch* pathway and *Tip60* mutation phenotypes. (A–D) Wings from heterozygotes or hemizygotes for alleles of the Notch pathway loci. (A) *Delta* (*Dl^BX9^*/*+*); (B) *notchoid* (*nd^1^*/*Y*); (C) *strawberry notch* (*sno^E1^*/*Y*); and (D) *mastermind* (*mam^N2G^*/*+*), each exhibits minor wing phenotypes. (F–I) When these genotypes were combined with *C96-domR*, the phenotypes exhibited significant enhancement: (F) *Dl^BX9^*/*C96-domR*; (G) *nd^1^*/*Y*; *C96-domR*/+; (H) *sno^E1^* /*Y*; *C96-domR/+*; and (I) *mam^N2G^*/*+*; *C96-domR/+*. (E) Wings from flies expressing *UAS-Tip60* RNAi across the margin under *C96-GAL4* regulation exhibit a wild-type phenotype. (J) When the *UAS-Tip60* RNAi construct is combined with *C96-domR*, very strong enhancement of the *C96-domR* wing phenotype is apparent.

We also tested *C96-domR* for other predicted genetic interactions. Prior studies (Hall *et al.* 2004; Eissenberg *et al.* 2005) and Cut and Wg expression data (Figures 2 and 3) predict an effect on *Notch*. Likewise, an interaction is predicted for LOF in loci encoding components of the Tip60 acetyltransferase complex, because Dom is a member of that complex (Kusch *et al.* 2004). Alleles of canonical Notch pathway loci (*Delta*, *Notch*, and *mastermind*) and *strawberry notch* (Figure 4, F–I) exhibit strong phenotypic enhancement of *C96-domR* relative to that of wings from outcrosses to *w^1118^* (Figure 4, A–D). Similarly, LOF for *Tip60* generated through RNAi exhibits no phenotype (Figure 4E). However, *Tip60* RNAi coexpression leads to strong enhancement of the *C96-domR* wing margin phenotype (Figure 4J). We tested mutations in several other loci that encode Tip60 complex components (Kusch *et al.* 2004) and observed a significant increase in wing nicking penetrance (*P* < 0.01) for alleles of *E*(*Pc*), *MRG15*, *TRA1*, and *rept*. Although the effects were not as strong as for *Tip60*, these data further validate the *domR* phenotype and also demonstrate that Tip60 complex components function at the wing margin.

### Screen for *dom* modifiers

**Figure 5:**
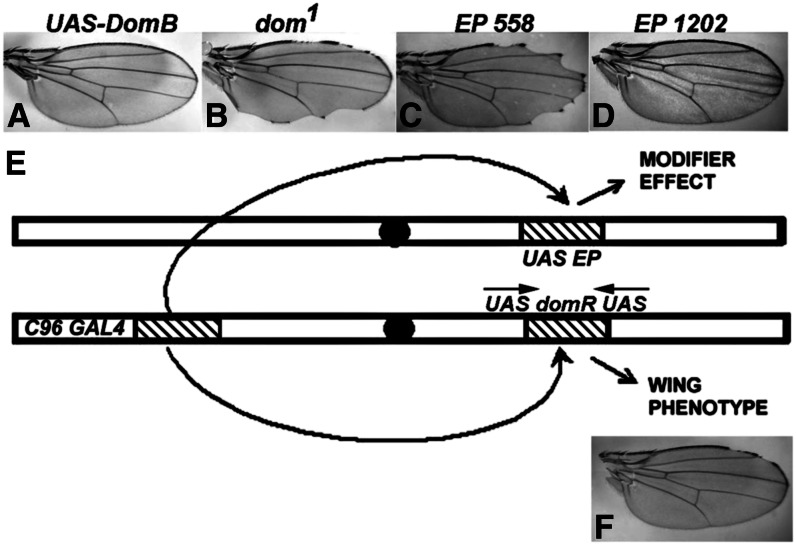
*dom* modifier screen. (E) shows the recombinant chromosome *C96-domR* containing the *C96-GAL4* and *UAS-domR* transgenes. It produces the weak wing nicking phenotype shown in (F; penetrance of ∼57%). (A and B) Controls demonstrating that the wing phenotype is affected by changes in the level of wild-type Dom expression, either through *GAL4* coexpression of a wild-type Dom construct [*UAS-DomB* (A)] or through a LOF allele [*dom^1^* (B)], as described in the text. The *EP* screen involves testing individual *EP* insertion chromosomes as transheterozygotes with *C96-domR* (E). In these genotypes, *C96-GAL4* can drive both *UAS-domR* and a random sequence if the *EP* insertion is oriented appropriately. The *EP* element also generates LOF alleles. Wing modifications, such as enhancement [*EP558* (C), 88% penetrance and increased severity] or suppression [*EP1202* (D), 0% penetrance, total suppression] are scored.

*C96-domR* heterozygosity creates a dominant but hypomorphic condition appropriate for genetic screening (Figure 2). We performed a screen for modifiers of this phenotype through mobilization of *EP*, a *P* transposon that creates both overexpression and LOF alleles (Rorth 1996; Thibault *et al.* 2004). *EP* elements were mobilized, and each insertion was tested for phenotypic effects when combined with *C96-domR*. The *C96-GAL4* element within *C96-domR* drives both *dom* RNAi and the sequence downstream of the *EP* insertion, potentially creating a modifier effect (Figure 5). For example, the wing phenotype of *C96-domR*/*w^1118^* (Figure 5F) can be either suppressed by overexpression of a normal Dom product (Figure 5A) or enhanced by a *dom* LOF allele (Figure 5B). Thus, the *C96-domR* wing phenotype allows detection of enhancers and suppressors. Figure 5, C and D, shows two modifiers from the screen, an enhancer (*EP558*) and a suppressor (*EP1202*).

**Figure 6:**
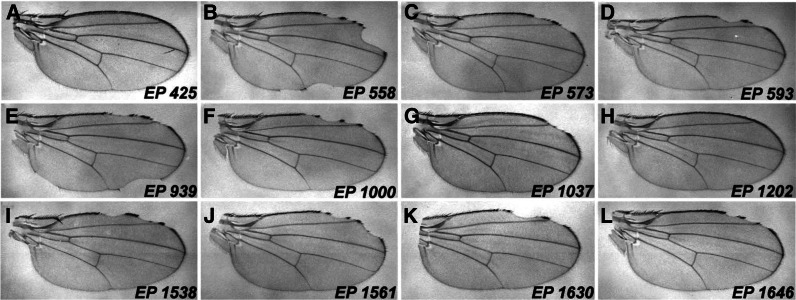
*EP* modifier effects on *C96-domR* wing phenotypes. Wing mounts were prepared from crosses of the *C96-domR* strain with the following *EP* modifiers: (A) *EP 425* (*tara*); (B) *EP 558* (*pabp2*); (C) *EP 573* (*Lk6*); (D) *EP 593* (*Tudor-SN*); (E) *EP 939* (*EcR*); (F) *EP 1000* (*lola*); (G) *EP 1037* (*wdb*); (H) *EP 1202* (*atg1*); (I) *EP 1538* (*lola*); (J) *EP 1561* (*emc*); (K) *EP 1630* (*lilli*); (L) *EP 1646* (*pum*). Note that the *EP*s are classified as enhancers or suppressors based upon the penetrance of wing nicking relative to that of *w^1118^* control crosses to *C96-domR*, rather than the severity of wing blade loss or nicking (see *Materials and Methods*). These data are summarized in Table 2.

We tested 2500 transpositions and focused on 12, 9 enhancers and 3 suppressors (Table 2). Several additional modifiers were identified through complementation tests as canonical Notch pathway components and not characterized further. None of the *EP* strains exhibits a wing phenotype alone or when crossed with *C96-GAL4*. This demonstrates that phenotypes derive from a synergistic interaction due to expression of *dom* RNAi and consequent loss of *dom* function. Inverse PCR and DNA sequencing were performed to identify the most proximal locus and determine the *EP* orientation relative to the coding region. Predicted overexpression (GOF) or insertional knockout (LOF) effects are shown in Table 2. One suppressor and three enhancers are oriented for a GOF effect. Table 2 lists these modifiers along with the affected loci, sites of insertion, and effects on the penetrance of the *C96-domR* phenotype. We validated the genetic interactions of *C96-domR* with independent alleles of the targeted loci. These alleles were either GOF or LOF, matching the predicted nature of the *EP* modifier. As shown in Table 3, *C96-domR* exhibits parallel genetic interactions with these strains and the original *EP*s. Figure 6 shows representative wings from *C96-domR* as transheterozygotes with the 12 *EP* insertions.

**Table 2:** Characterization of modifier alleles

EP#	Gene	Insertion Site (position)	Mutation	*C96-domR*	*N* Wings Scored	*C96-MamH*	*N* Wings Scored
*425*	*tara*	Intron (12075314)	LOF	S (5%)	414	S+	92
*558*	*pabp2*	Exon (4019484)	LOF	E (88%)	312	E	100
*573*	*Lk6*	Intron (7585856)	LOF	E (68%)	1023	S	206
*593*	*Tudor-SN*	Upstream (264378)	GOF	E (74%)	426	ne	288
*939*	*EcR*	Intron (2007989)	LOF	E (88%)	336	E	110
*1000*	*lola*	Not Determined	LOF	E (91%)	251	E+	100
*1037*	*wdb*	Intron (23402526)	GOF	S (20%)	368	S+	98
*1202*	*atg1*	Exon (12798085)	LOF	S (0%)	338	S+	90
*1538*	*lola*	Intron (6421948)	LOF	E (91%)	330	E+	86
*1561*	*emc*	Upstream (749363)	GOF	E (85%)	240	S+	130
*1630*	*lilli*	Intron (2900668)	GOF	E (81%)	378	E+	194
*1646*	*pum*	Intron (4983814)	LOF	E (70%)	250	E+	130

The percentage of wing nicking is shown for crosses of *C96-domR* to *EP* modifiers in column labeled *C96-domR*. All wing nicking differences were highly significant (*P* < 0.001, chi-square test relative to *w^1118^* control; 57%, Table 1). All *EP* tests included *w^1118^* controls, and wing nicking percentages were normalized to 57% control value between experiments. *EP 1000* was determined to be an allele of *lola* through a complementation test. The *C96-MamH* genotype produces 100% wing nicking when outcrossed to *w^1118^* control. Among this control class we determined the percent of wings with weak, moderate, and strong effects including extent of nicks and blade loss. We then compared the distribution of severity in the *EP* cross progeny to determine if there was suppression (S), strong suppression (S+), enhancement (E), strong enhancement (E+) or no effect (ne); these data are presented in column labeled *C96-MamH*. GOF, gain of function; LOF, loss of function.

**Table 3:** Corroboration of *EP* insertion alleles

*EP*#	Corroborating Allele	*C96-domR* (% of Nicking)	*N* Wings Scored
*425*	*tara^1^*	S (6%)	300
*558*	*pabp2^01^*	E (96%)	361
*573*	*LK6^2^*	E (95%)	594
*593*	*P(EP^g42^)Tudor-SN^Ey07875^*	E (79%)	736
*939*	*EcR^M554fs^*	E (81%)	144
*1000*	*lola^ORE119^*	E (97%)	114
*1037*	*UAS-Wdb*	S (48%)	620
*1202*	*unc51^3^ (atg1)*	S (36%)	1126
*1538*	*lola^ORE119^*	E (97%)	114
*1561*	*P{EPgy2}emc^EY01657^*	E (83%)	262
*1630*	*P{EPgy2}lilli^EY11976^*	E (100%)	283
*1646*	*1(3)pum^01688^*	E (90%)	392

The interaction between the corroborating allele for each *EP* and *C96-domR* is as described for Table 2. All wing nicking differences *vs. w^1118^* control (57%) were highly significant (*P* < 0.001, chi-square test). For the GOF alleles *EPs 1630*, *1561*, *593*, and *1037*, we corroborated with overexpression strains. E, enhancement; S, suppression.

The 12 modifiers encode three classes of functions. The first two classes include transcription factors [*taranis* (*tara*), *lilliputian* (*lilli*), *longitudinals lacking* (*lola*), *Ecdysone Receptor* (*EcR*), and *extramacrochaete* (*emc*)] and proteins that regulate RNA function, including *pumilio* (*pum*), *polyA binding protein 2* (*pabp2*), and *Tudor-SN*. Members of both classes have been associated with wing formation and/or Notch signaling previously (Kankel *et al.* 2007; Shalaby *et al.* 2009). The third class encodes proteins linked to cell growth and autophagy pathways, and includes the Ser/Thr protein phosphatase (PP2A) regulator *widerborst* (*wdb*; Vereshchagina *et al.* 2008), the protein kinase *Lk6* (Arquier *et al.* 2005), and *atg1*, a regulator of autophagy, which is a lysosomal degradation pathway that affects cell growth (Zirin and Perrimon 2010).

Table 2 also summarizes interactions between the *EP* modifiers and a strain that drives truncated Mastermind across the wing margin (*C96-MamH*), which creates a Notch pathway LOF phenotype. Expression of truncated Mastermind has been shown to depress Notch signaling in multiple contexts including the wing margin (Helms *et al.* 1999), where it leads to a 100% penetrant nicking phenotype. Most of the *EP* strains exhibit similar interactions between *C96-domR* and *C96-MamH* as enhancers or suppressors, as expected. However, for the case of the *C96-domR* enhancer *EP 1561* (*emc*) there was strong suppression of *C96-MamH*. This was validated with the canonical GOF allele (*emc^D^*), which also suppressed, and two LOF *emc* alleles, which enhanced (data not shown). The *C96-domR* enhancer *EP 573* (*Lk6*) slightly suppressed *C96-MamH*. However, the *Lk6^2^* LOF allele enhanced *C96-MamH*, matching its effect on *C96-domR*. The *C96-domR* enhancer strain *EP 593* (*Tudor-SN*) did not affect *C96-MamH*, possibly reflecting activity directed at RNAi processing (see *Discussion*).

### *dom* wing phenotype is sensitive to changes in growth and autophagy loci

The *atg1* (*unc51*) gene regulates autophagy and growth pathways in numerous organisms including *Drosophila* (Zirin and Perrimon 2010). The *EP 1202* (*atg1*) modifier allele, as well as its corroborating *unc51^3^* allele (Toda *et al.* 2008), each behave as strong suppressors of *C96-domR* (Tables 2 and 3), indicating a link of *dom* function to these processes. Likewise, the *EP 573* allele of *Lk6* and the corroborating *Lk6^2^* allele (Tables 2 and 3) are both strong enhancers of the *C96-domR* phenotype. Lk6 is related to mammalian kinases that regulate cell growth and division (Arquier *et al.* 2005). Finally, *EP 1037* and the corroborating GOF *UAS-Wdb* strain are suppressors of *C96-domR* (Tables 2 and 3). Wdb, a regulatory subunit of PP2A has been associated with both cell growth regulation and autophagy (Arquier *et al.* 2005, Vereshchagina *et al.* 2008; Banreti *et al.* 2012). The isolation of three modifiers associated with these processes suggested that loss of *dom* function may enrich for this class of loci.

Table 4 shows data from crosses of *C96-domR* with strains expressing RNAi directed against 10 different autophagy pathway loci, as well as one strain that overexpresses the normal *atg1* product. Seven of the 10 assayed *atg* genes enhance the *C96-domR* wing phenotype when their function is depressed, indicating that normal autophagy activity can limit wing margin loss derived from depressed *dom* function (*atg6*, *atg7*, *atg8A*, *atg8B*, *atg12*, *atg9*, and *atg5*). Three RNAi strains, *atg2*, *atg4* , and *atg18* , do not show a significant effect, and these loci encode various functions within the autophagy pathway (Chang and Neufeld 2010). It is possible that these strains do not effectively down-regulate their target loci or that, alternatively, there may be genetic redundancy for certain loci. The enhancement effect of multiple *atg* RNAi strains contrasts with the *atg1* effect, where LOF was observed to suppress *C96-domR* (Tables 2 and 3). Moreover, overexpression of *atg1* across the wing margin strongly enhances the wing phenotype (Table 4), consistent with the LOF suppression effect. The differential effects of *atg1 vs.* other *atg* genes likely reflect the additional roles of *atg1* in translational efficiency and growth regulation (Lee *et al.* 2007), beyond its role in autophagy regulation (see *Discussion*).

**Table 4:** Additional genetic interactions of *dom*

Genotype	*C96-domR* (% of Nicking)	*N* Wings Scored
*UAS- atg1 (Unc51)*	E (86%)	322
*atg6, TRiP*	E (82%)	2074
*atg7, TRiP*	E (75%)	1724
*atg8A, TRiP*	E (73%)	1645
*atg8B, TRiP*	E (78%)	2227
*atg12, TRiP*	E (73%)	2147
*atg9, TRiP*	E (72%)	1520
*atg5, TRiP*	E (63%)	1773
*atg4, TRiP*	ne (58%)	2049
*atg2, TRiP*	ne (58%)	1736
*atg18, TRiP*	ne (55%)	1845
*PP2A wdb^7^* Regulatory	E (70%)	638
*PP2A wdb, TRiP* Regulatory	E (91%)	236
*PP2A tws, TRiP* Regulatory	E (83%)	1021
*PP2A wrd, TRiP* Regulatory	E (68%)	1748
*PP2A mts,* TRiP Catalytic*^a^*	E (100%)	478
*PP2A 29B,* TRiP Scaffold*^a^*	E (100%)	350
*CK1, UAS-dcoK4*	E (90%)	1028
*CK1, dco TRiP*	S (40%)	1390

Column labeled *C96-domR* shows the percentage of wing nicking from crosses of *C96-domR* to listed genotypes. All genotypes are *UAS*-regulated except for *wdb^7^*. The interaction between these genotypes and *C96-domR* is as described for Table 2. All wing nicking differences were highly significant (*P* < 0.001, chi-square test), except for those labeled ne (no effect). Regulatory, catalytic and scaffold indicate the encoded function within the PP2A phosphatase complex. E, enhancer; S, suppressor.

a These strains exhibited wing nicking at high penetrance with control crosses to *C96-GAL4*; all other *UAS* strains showed normal wings in the control cross.

The *EP 1037* and corroborating *UAS-Wdb* strains appear to suppress *C96-domR* through overexpression of Wdb, a regulatory subunit of the PP2A phosphatase (Tables 2 and 3). We examined other strains with LOF for either *wdb* or other regulatory, scaffold and catalytic subunits of the PP2A complex, and observed enhancement of *C96-domR* (Table 4). This is consistent with the suppression derived from Wdb overexpression. These data predict that a class of Ser/Thr kinases may act antagonistically to PP2A phosphatase. *Casein kinase 1* (*Ck1*) overexpression strongly enhances the *C96-domR* wing phenotype, whereas LOF suppresses (Table 4). These effects are opposite to those derived from alterations in PP2A phosphatase. PP2A and CK1 have been shown to act antagonistically in other developmental contexts (Jia *et al.* 2009).

### *dom* and its modifiers interact genetically with a proliferation-defective genotype

**Figure 7:**
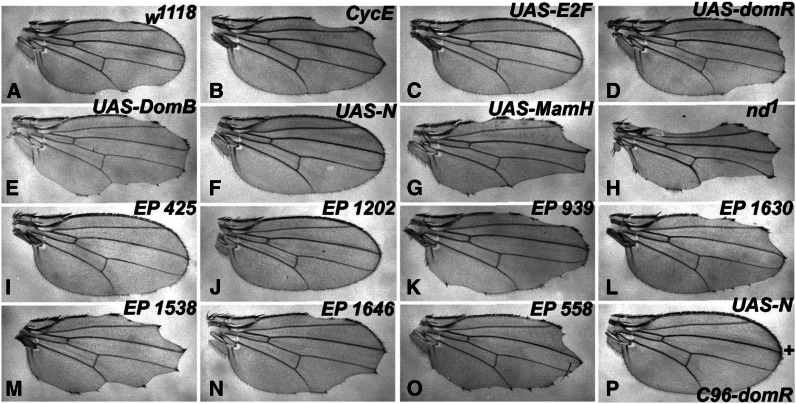
Interactions of the *C96+Rbf280* proliferation-defective genotype. In the descriptions below, numbers in parentheses indicate percentage of nicked wings and number of wings scored (N). (A) (30%, N = 440) shows that a constitutively active form of Rbf (Rbf-280) driven across the wing margin via *C96-GAL4* (*UAS-Rbf-280 + C96-GAL4*/*+*, referred to here as *C96+Rbf280*) creates a partially penetrant, dominant wing nicking phenotype as a heterozygote with *w^1118^*. (B–O) Wings transheterozygous with *C96+Rbf280*. (B) (64%, N = 120) shows an enhanced wing phenotype via combination with *cycE^AR95^*; (C) (18%, N = 86) shows partial rescue in combination with *UAS-E2F*. Both the *UAS-domR* RNAi transgene (D) (77%, N = 164) and the *UAS-DomB* transgene (E) (68%, N = 166) elicit strong enhancement. *UAS-DomB* did not elicit wing nicking in control crosses to *C96-GAL4*, data not shown (0%, N = 120). (F) (2%, N = 360) Nearly complete suppression of the wing phenotype by coexpression of an activated Notch construct, *UAS-N*, whereas depressed Notch signaling via *UAS-MamH* (G) (100%, N = 174) or the hemizygous viable *nd^1^* allele (H) (100%, N = 274) strongly enhance. (I–O) *C96+Rbf280* wings transheterozygous with *EP* modifiers from the screen: *EP 425 tara* (I) (10%, N = 124), *EP 1202 atg1* (J) (2%, N = 56), *EP 939 EcR* (K) (92%, N = 52), *EP 1630 lilli* (L) (70%, N = 146), *EP 1538 lola* (M) (98%, N = 50), *EP 1646 pum* (N) (72%, N= 116), *EP 558 pabp2* (O) (80%, N = 54). (P) (0%, N = 253) Complete suppression of the *C96-domR* wing nicking by coexpression of the activated Notch construct *UAS-N*. See Figure 2B legend for *C96-domR* wing phenotype. The penetrance of wing nicking in panels B–P is significantly different than *w^1118^* control crosses (*P* < 0.01, chi-square test).

*Dom* has been implicated in the regulation of cell proliferation (Braun *et al.* 1997; Lu *et al.* 2007). Therefore, we generated a strain with a dominant proliferation-defective phenotype at the wing margin, and tested for genetic interaction with *C96-domR* and its modifiers. The test utilizes a mutated version of the Rbf protein (Rbf-280), where four Cdk phosphorylation sites have been inactivated. This results in a constitutively active form of Rbf that blocks growth and proliferation in wing tissue (Xin *et al.* 2002). We found that when *UAS-Rbf280* is driven by *C96-GAL4* at the margin (*C96+Rbf280*), it elicits a dominant, partially penetrant wing nicking phenotype and loss of a subset of anterior margin bristles (Figure 7A). As predicted for a proliferation defect, this phenotype is enhanced by a mutation in *cycE* and suppressed by overexpression of E2F (Figure 7, B and C). The combination of *C96+Rbf280* with *UAS-domR* leads to enhanced penetrance and more severe loss of wing material (Figure 7D); enhancement was also observed with the canonical *dom^1^* and *dom^3^* alleles to a significant but lesser extent (data not shown).

Paradoxically, we also observed *C96+Rbf280* enhancement when Dom protein was overexpressed across the wing margin via the *UAS-DomB* transgene (Figure 7E). These results likely reflect pleiotropy of Dom function, because it is necessary for both Notch target expression (Figure 2) and repression of proliferation (Lu *et al.* 2007). *C96-domR* RNAi-mediated down regulation of Notch signaling at the wing margin is predicted to depress proliferation (Baonza and Garcia-Bellido 2000; Ferres-Marco *et al.* 2006) and thereby enhance the *C96+Rbf280* phenotype. Consistent with this prediction, coexpression of activated Notch via *UAS-N* rescued nearly completely the *C96+Rbf280* proliferation defect (Figure 7F); whereas depressions in Notch signaling strongly enhanced it (Figure 7, G and H). In contrast, Dom can also act as an inhibitor of cell proliferation through repression of E2F target genes (Lu *et al.* 2007), and overexpression via *UAS-DomB* may act primarily by enhancing the cell proliferation-defective phenotype of *C96+Rbf280*. The effect of *dom* LOF on Notch signaling is also evident by complete rescue of the *C96-domR* phenotype through simultaneous expression of *UAS-N* (Figure 7P).

Two of the *C96-domR* suppressors, *EP*s *425* (*tara*) and *1202* (*atg1*), were found to be strong suppressors of *C96+Rbf280* (Figure 7, I and J). Likewise, several of the *C96-domR* enhancers were also found to strongly enhance *C96+Rbf280*, including *EP*s *939* (*EcR*), *1630* (*lilli*), *1538* (*lola*), *1646* (*pum*), and *558* (*pabp2*) (Figure 7, K–O). The remaining modifiers showed much weaker interactions (data not shown).

## Discussion

We have described a screen for loci that interact with *dom* at the wing margin. Based on the intersection of *dom* with Notch signaling, we expected to identify a broad array of loci, and most of the *dom* modifiers interact similarly with a strain defective in Notch signaling. Additionally, based on the association of both *Notch* and *dom* with cell proliferation (Braun *et al.* 1997; Baonza and Garcia-Bellido 2000; Ferres-Marco *et al.* 2006; Lu *et al.* 2007), it is not surprising that most of the modifiers have been linked to cell growth and division, and exhibit genetic interaction with a proliferation-defective genotype. Moreover, as LOF for *dom* has also been associated with cell death (Braun *et al.* 1997), it is possible that this process also contributes to the phenotypes we describe here. Wing margin staining for key markers associated with cell death *vs.* cell cycling will be necessary to establish the basis for these effects. Nevertheless, the *C96-domR* phenotype appears pleiotropic, derived from effects on Notch signaling, growth, proliferation and likely other factors operating at the wing margin.

Several modifiers encode transcription factors, such as *lilli*, *tara*, and *emc*, which were identified in a prior screen targeted to the wing (Kankel *et al.* 2007). Lilli, a protein of the fragile X/Burkitt’s lymphoma class (Su *et al.* 2001), was also linked to wing margin formation by Bejarano *et al.* (2008). *Tara*, a member of the trithorax group, was isolated in a screen for modifiers of a homeotic phenotype (Calgaro *et al.* 2002). *Tara* functions opposite to *dom* with homeotic loci (Ruhf *et al.* 2001; Calgaro *et al.* 2002). Antagonism between *tara* and *dom* is consistent with our observation that loss of *tara* suppresses the wing nicking derived from loss of *dom*. The Tara protein shows sequence similarity to transcriptional regulators of cell cycle proteins (Calgaro *et al.* 2002), and it is noteworthy that *tara* mutation also suppresses wing nicking associated with the proliferation-defective *C96+Rbf280* strain (Figure 7C). *Emc*, a negative regulator of HLH transcription factors, has complex functions at the wing margin, affecting both cell proliferation and sensory organ formation (Baonza *et al.* 2000). Thus, GOF for *emc* through *EP 1561* may enhance margin effects through suppression of sensory bristle formation.

Lola, related to the broad complex class of transcription factors is required for central nervous system development (Giniger *et al.* 1994) and wing margin patterning through an interaction with *cut* (Krupp *et al.* 2005). Lola has also been implicated in cell proliferation and oncogenesis through Notch-mediated repression of *Rbf* (Ferres-Marco *et al.* 2006). Therefore, LOF alleles may derepress Rbf expression and inhibit cell proliferation. This is consistent with our observation that *lola* alleles enhance the *C96-domR* and *C96+Rbf280* strains (Figures 6 and 7). Finally, ecdysone receptor (EcR) function is associated with sensory organ differentiation (Schubiger *et al.* 2005), and Dom was identified as an EcR cofactor in cultured cells (Davis *et al.* 2011).

A second class of *dom* modifier encodes RNA regulatory proteins. Pabp2 regulates polyA tail length, and LOF of *pabp2* is associated with aberrant levels of Cyclin B (Benoit *et al.* 2005). Pumilio is an RNA-binding protein that mediates translational repression (Wharton *et al.* 1998). Additionally, loss of Pumilio function has been associated with improper regulation of Cyclin B (Sonoda and Wharton 2001). Consistent with a cell cycle defect, *EP*s *558* (*pabp2*) and *1646* (*pum*) strongly enhance the *C96+Rbf280* wing phenotype (Figure 7). Tudor-SN has been implicated in transcription, processing, and RNA interference as a subunit of the RNA-induced silencing complex (Friberg *et al.* 2009). The *Tudor-SN* overexpression alleles *EP 593* and *P*(*EP^g42^*)*Tudor-SN^Ey07875^* (Tables 2 and 3) could act through enhanced production of *dom* RNAi. The observation that *EP 593* does not modify *C96-MamH* or *C96+Rbf280* phenotypes, which are both produced independently of RNAi, supports this idea.

The third class of modifier links *dom* to antagonistic growth and autophagy pathways. During growth, the degradation of organelles and long-lived proteins associated with autophagy is suppressed through inactivation of Atg1, a serine/threonine kinase (Zirin and Perrimon 2010). In contrast, during conditions of cellular starvation or stress Atg1 is not suppressed. It is required for autophagy induction, thereby providing raw materials for cell survival. However, Atg1 has additional functions, including the down-regulation of growth through inactivation of S6 kinase. The S6 kinase normally phosphorylates ribosomal protein S6, and this activity is a hallmark of cell growth (Lee *et al.* 2007). Thus, Atg1 functions at a key juncture, to both induce autophagy and prevent cell growth under conditions inappropriate for growth. Our data demonstrates that depressed *atg1* function suppresses the *C96-domR* phenotype, whereas overexpression enhances it. Mutation of *atg1* should lead to elevations in S6 kinase activity (Lee *et al.* 2007) and increases in translation and cell division, and this could mediate the wing margin rescue we observe.

Concomitantly, rescue derived from *atg1* mutation should be associated with depressed autophagy induction. This *atg1* effect would contradict our data on seven other *atg* loci, where LOF enhances the wing phenotype, rather than suppresses. The data from these seven loci suggest that normal levels of autophagy act to limit wing margin loss associated with *C96-domR*. This could occur, for example, if cells interpret *dom* loss as stress and launch autophagy as a response to provide the raw materials for repair. These conflicting data can be reconciled if, in the case of *atg1* mutation, the resultant growth elevation is epistatic to effects of autophagy depression. During conditions favoring growth and cell division, autophagy may no longer be required for wing margin rescue. Our observation that *EP 1202* (LOF *atg1*) suppresses the wing defects derived from both *C96-domR* and *C96+Rbf280* favors this idea (Figures 6 and 7). The association of *dom* with *atg* mutations is consistent with an earlier report linking autophagy to Notch signaling in the wing (Thumm and Kadowaki 2001).

Additional effects of *dom* loss on autophagy may contribute to the wing margin phenotype. Mammalian Tip60 protein, upon phosphorylation and activation by the GSK3 kinase, acetylates and activates Atg1 (ULK1) during autophagy induction (Lin *et al.* 2012). This reveals a role for Tip60 acetyltransferase directly in autophagy regulation, rather than through genetic regulation. However, it is not known if this mechanism operates in *Drosophila* and, if it does, whether Dom or the remainder of the Tip60 complex also plays a role.

A second link to growth and autophagy pathways derived from our screen was *wdb*, which encodes a protein phosphatase (PP2A) regulatory subunit. Wdb regulates many functions, including protein kinase activity and growth (Vereshchagina *et al.* 2008). Wdb was also implicated as a positive regulator of autophagy in *Drosophila*, targeting several Atg proteins (Banreti *et al.* 2012). One of the postulated targets is Atg1, which has been shown to be a PP2A target in *C. elegans* (Ogura *et al.* 2010). Alternatively, Wdb could covalently modify Dom protein and alter its activity. Wdb has been associated with Hedgehog signaling through dephosphorylation and down regulation of the cubitus interruptus protein (Jia *et al.* 2009). That study showed opposite effects mediated by PP2A phosphatase *vs.* CK1 kinase, similar to our observations with the *C96-domR* phenotype (Table 4). Although we have no evidence supporting such modifications, the predicted Dom sequence contains consensus sites for CK1 phosphorylation (data not shown).

Finally, we found that mutations in the *Lk6* locus affect the *C96-domR* phenotype (Tables 2 and 3). The *Drosophila* Lk6 protein is the functional homolog of mammalian Mnk kinases, which regulate the activity of translational initiation factor eIF4E and growth through phosphorylation. Mutation of *Lk6* has been associated with organismal growth depression and reduced wing size through reduced cell number (Arquier *et al.* 2005). Contrasting effects of Lk6 on growth in *Drosophila* have also been reported, dependent on nutrient levels (Reiling *et al.* 2005), indicating that regulation of Lk6 is sensitive to culture conditions.

These links of *dom* to growth and autophagy are likely related to its effects on Notch signaling and cell proliferation, with some contribution due to a cell death effect also plausible. Our results (Figures 2 and 7), in conjunction with prior reports, indicate a Dom requirement for both Notch target expression and repression of cell proliferation (Hall *et al.* 2004; Eissenberg *et al.* 2005; Gause *et al.* 2006; Lu *et al.* 2007). Together, these results suggest a model where Dom contributes to positive regulation of Notch signaling, which in turn stimulates cell proliferation. Dom is subsequently involved in negative regulation of proliferation through repression of E2F-dependent loci. Although this model is consistent with most data, other work has implicated Dom as a positive effector during proliferation. Larvae homozygous for the most severe *dom* alleles were observed to be lacking imaginal discs and exhibited a reduction in brain neuroblasts (Ruhf *et al.* 2001), and recently a study of larval tissues showed that Dom is required for expression of several cell cycle loci, including the *cyclins E*, *B*, *B3*, *A*, *CDC2*, and *string* (Walker *et al.* 2011). Additionally, Lu *et al.* (2007) observed that Dom is resident at the promoter of numerous E2F target loci required for cell proliferation. However, contrary to its role in proliferation inhibition, they found that reduction of Dom levels is not associated with elevated expression of various S-phase loci, including *cyclin E*. An interesting possibility is that Dom functions at both inactive and active E2F target promoters, potentially contributing to a switch between negative and positive regulation of transcription and cell division. Notch signaling could contribute to the switch, in the same manner that the canonical pathway regulates targets such as *E*(*spl*) loci, through the displacement of a repression complex and recruitment of transcriptional activators (Lubman *et al.* 2007). Under such circumstances, the phenotypic consequences of altered Dom levels could vary significantly, as previously observed (Braun *et al.* 1997; Ruhf *et al.* 2001; Lu *et al.* 2007; Walker *et al.* 2011). Additional biochemical assays of Dom and Notch function will be necessary to address such possibilities.

In conclusion, we found that targeting LOF for *dom* to the wing margin created a sensitized genotype associated with a partially penetrant, dominant phenotype. This phenotype was scored for dosage-sensitive modifiers, allowing an efficient scan for other loci that function with *dom*. Our analysis demonstrated that *dom* modifiers are enriched for loci that contribute to the regulation of cell growth and proliferation, which is consistent with prior studies of *dom* function. This genetic system will facilitate screening for novel loci involved with growth regulatory mechanisms, and complement biochemical approaches to the same questions.
